# High-quality surrounding landscapes mitigate avian extirpations from forest remnants

**DOI:** 10.1073/pnas.2521783123

**Published:** 2026-04-01

**Authors:** Anderson S. Bueno, Chase D. Mendenhall, Marina Anciães, Luiz dos Anjos, Víctor Arroyo-Rodríguez, Marco Aurélio-Silva, Cristina Banks-Leite, Matthew G. Betts, Arthur A. Bispo, Andrea Larissa Boesing, Marconi Campos-Cerqueira, Olivier Claessens, Will Cresswell, Gretchen C. Daily, Filibus Danjuma Dami, Sidnei M. Dantas, Pedro F. Develey, Ping Ding, David P. Edwards, Márcio A. Efe, Deborah Faria, Kenneth J. Feeley, Thomas W. Gillespie, Adam S. Hadley, Jack H. Hatfield, Luiza Magalli Pinto Henriques, Lars H. Holbech, Gregory J. Irving, Urs G. Kormann, M. Jyothi Krishnan, Marilise M. Krügel, Jesse R. Lasky, Michael J. Lawes, Alexander C. Lees, Luc Lens, Lahert William Lobo-Araújo, Leithen K. M’Gonigle, Mohammad Saiful Mansor, Shiiwua A. Manu, Miguel Ângelo Marini, Alexandre Camargo Martensen, Thomas J. Matthews, Jean Paul Metzger, Randall Moore, José Carlos Morante-Filho, P. O. Nameer, Shukor Md Nor, Helon Simões Oliveira, Rômulo Ribon, Viviana Ruiz-Gutiérrez, Luís Fábio Silveira, Philip C Stouffer, John W. Terborgh, Alexandre Uezu, Yanping Wang, Robyn Wethered, Ding Li Yong, Carlos A. Peres

**Affiliations:** ^a^Instituto Federal de Educação, Ciência e Tecnologia Farroupilha, Júlio de Castilhos, RS 98130-000, Brazil; ^b^Physician Assistant Studies, Slippery Rock University, Slippery Rock, PA 16057; ^c^Instituto Nacional de Pesquisas da Amazônia, Manaus, AM 69067-375, Brazil; ^d^Departamento de Biologia Animal e Vegetal, Universidade Estadual de Londrina, Londrina, PR 86057-970, Brazil; ^e^Instituto de Investigaciones en Ecosistemas y Sustentabilidad, Universidad Nacional Autónoma de México, Morelia 58190, Mexico; ^f^Escuela Nacional de Estudios Superiores, Universidad Nacional Autónoma de México, Mérida 97357, Mexico; ^g^Department of Life Sciences, Imperial College London, Ascot SL5 7PY, United Kingdom; ^h^Department of Forest Ecosystems and Society, Oregon State University, Corvallis, OR 97331; ^i^Laboratório de Etnobiologia e Biodiversidade, Universidade Federal de Goiás, Goiânia, GO 74690-900, Brazil; ^j^Departamento de Ecologia, Universidade de São Paulo, São Paulo, SP 05508-090, Brazil; ^k^Senckenberg Biodiversity and Climate Research Centre, Frankfurt am Main 60325, Germany; ^l^WildMon, Lewes, DE 19958; ^m^Groupe d’Étude et de Protection des Oiseaux en Guyane, Rémire-Montjoly 97354, French Guiana, France; ^n^Centre for Biological Diversity, University of St. Andrews, St. Andrews KY16 9TH, United Kingdom; ^o^Department of Biology, Stanford University, Stanford, CA 94305; ^p^A.P. Leventis Ornithological Research Institute, University of Jos, Jos 930001, Nigeria; ^q^Instituto Tecnológico Vale, Belém, PA 66055-090, Brazil; ^r^SAVE Brasil, São Paulo, SP 05427-010, Brazil; ^s^College of Life Sciences, Zhejiang University, Hangzhou 310058, China; ^t^Department of Plant Sciences and Centre for Global Wood Security, University of Cambridge, Cambridge CB2 3EA, United Kingdom; ^u^Laboratório de Bioecologia e Conservação de Aves Neotropicais, Universidade Federal de Alagoas, Maceió, AL 57072-900, Brazil; ^v^Departamento de Ciências Biológicas, Universidade Estadual de Santa Cruz, Ilhéus, BA 45662-900, Brazil; ^w^Department of Biology, University of Miami, Coral Gables, FL 33146; ^x^Department of Geography, University of California, Los Angeles, CA 90095; ^y^New Brunswick Department of Natural Resources and Energy Development, Fredericton, NB E3B 5H1, Canada; ^z^Leverhulme Centre for Anthropocene Biodiversity and Department of Biology, University of York, York YO10 5DD, United Kingdom; ^aa^Department of Animal Biology and Conservation Science, University of Ghana, P.O. Box LG 67, Legon, Accra, Ghana; ^bb^Conservation Ecology Program, King Mongkut’s University of Technology Thonburi, Bangkok 10150, Thailand; ^cc^Swiss Ornithological Institute, Sempach 6204, Switzerland; ^dd^Department of Wildlife Science, Kerala Agricultural University, Thrissur 680656, India; ^ee^Departamento de Engenharia Sanitária e Ambiental, Universidade Federal de Santa Maria, Santa Maria, RS 97105-900, Brazil; ^ff^Department of Biology, Pennsylvania State University, University Park, PA 16802; ^gg^School of Life Sciences, University of KwaZulu-Natal, Scottsville 3209, South Africa; ^hh^Institute of Biodiversity and Environmental Conservation, Universiti Malaysia Sarawak, Kota Samarahan 94300, Malaysia; ^ii^Department of Natural Sciences, Manchester Metropolitan University, Manchester M1 5GD, United Kingdom; ^jj^Cornell Lab of Ornithology, Cornell University, Ithaca, NY 14850; ^kk^Centre for Research on Ecology, Cognition and Behaviour of Birds, Ghent University, Ghent 9000, Belgium; ^ll^Programa de Pós-Graduação em Ecologia e Recursos Naturais, Universidade Federal de São Carlos, São Carlos, SP 13565-905, Brazil; ^mm^Department of Biological Sciences, Simon Fraser University, Burnaby, BC V5A 1S6, Canada; ^nn^Department of Biological Sciences and Biotechnology, Faculty of Science and Technology, Universiti Kebangsaan Malaysia, Bangi 43600, Malaysia; ^oo^Departamento de Zoologia, Universidade de Brasília, Brasília, DF 70910-900, Brazil; ^pp^Centro de Ciências da Natureza, Universidade Federal de São Carlos, Buri, SP 18299-000, Brazil; ^qq^School of Geography, Earth and Environmental Sciences and Birmingham Institute of Forest Research, University of Birmingham, Birmingham B15 2TT, United Kingdom; ^rr^Centre for Ecology, Evolution and Environmental Changes/Azorean Biodiversity Group/Global Change and Sustainability Institute and Universidade dos Açores–Faculty of Agricultural Sciences and Environment, Angra do Heroísmo, Açores 9700-042, Portugal; ^ss^Department of Fisheries, Wildlife, and Conservation Sciences, Oregon State University, Corvallis, OR 97331; ^tt^Programa de Pós-Graduação em Ecologia e Conservação, Universidade Federal de Sergipe, São Cristóvão, SE 49107-230, Brazil; ^uu^Museu de Zoologia João Moojen, Departamento de Biologia Animal, Universidade Federal de Viçosa, Viçosa, MG 36570-900, Brazil; ^vv^Museu de Zoologia da Universidade de São Paulo, São Paulo, SP 04263-000, Brazil; ^ww^School of Renewable Natural Resources, Louisiana State University AgCenter and Louisiana State University, Baton Rouge, LA 70803; ^xx^Department of Biology and Florida Museum of Natural History, University of Florida, Gainesville, FL 32611; ^yy^Centre for Tropical Environmental and Sustainability Science and College of Science and Engineering, James Cook University, Cairns, QLD 4870, Australia; ^zz^Instituto de Pesquisas Ecológicas, Nazaré Paulista, SP 12964-022, Brazil; ^aaa^Laboratory of Island Biogeography and Conservation Biology, College of Life Sciences, Nanjing Normal University, Nanjing 210023, China; ^bbb^Cossypha Ecological, Westville 3629, South Africa; ^ccc^BirdLife International (Asia), Tanglin International Centre, Singapore 247672, Singapore; ^ddd^School of Environmental Sciences, University of East Anglia, Norwich NR4 7TJ, United Kingdom; ^eee^Center for Biodiversity and Global Change, Yale University, New Haven, CT 06511; ^fff^Instituto Juruá, Manaus, AM 69057-060, Brazil

**Keywords:** biodiversity conservation, biogeography, habitat fragmentation, species richness, tropical forests

## Abstract

The species–area relationship (SAR) has long been used to predict extirpation rates from habitat loss, but these rates depend not only on habitat area but also on the surrounding landscape and species’ habitat specialization. We collated global data from forest islands created by river damming and forest fragments resulting from clear-cut deforestation to examine the effects of matrix type (aquatic or terrestrial) and tree cover on avian SARs. Unlike oceanic islands, which are often millions of years old, anthropogenic forest islands provide a contemporary analog to forest fragments to understand matrix effects on SARs and serve as a baseline for worst-case scenarios of forest fragmentation. Our database comprises 50 datasets from 45 studies conducted in tropical and subtropical regions, totaling 1,954 bird species detected through 39,197 incidence records from 336 forest islands and 669 forest fragments. We found that bird extirpation rates were lower in fragments than on islands, especially for forest-dependent species compared to all species. Species losses were further reduced by increasing tree cover around forest remnants at local landscape scales of 300 m, highlighting the importance of small-scale conservation strategies. Moreover, even small forest fragments with greater nearby tree cover held high conservation value, emphasizing the crucial role of the surrounding landscape in mitigating avian extirpations from forest remnants. Beyond protecting forest remnants themselves, area-based conservation efforts would therefore be greatly enhanced by improving matrix quality and expanding tree cover in otherwise hostile landscapes.

## Significance

In fragmented forest landscapes, species richness declines with decreasing forest remnant size, but the magnitude of these declines depends on the quality of the surrounding landscape and species’ habitat specialization. By examining the effects of matrix type and tree cover near forest remnants on species–area relationships, we found that forest fragments in terrestrial matrices supported more bird species than similar-sized forest islands surrounded by water. Species richness also increased with tree cover within 300 m of forest remnants, with stronger effects for forest-dependent species compared to all species. These findings indicate that the conservation value of forest remnants could be boosted by enhancing matrix quality—making it less hostile, more permeable, and richer in resources—and expanding nearby tree cover.

Biodiversity loss is a pressing challenge, with species extinctions and extirpations irreversibly altering ecosystems and threatening the many benefits they provide to society (1). The magnitude of this biodiversity crisis is daunting, with nearly nine thousand terrestrial vertebrate species showing signs of decline (2). The foremost strategies to mitigate this ongoing mass decline in species are area based, including sparing wildlife habitat by improving intensive farming practices (3) and setting aside large interconnected protected areas (4). A complementary strategy is conserving biodiversity in human-modified landscapes—such as pasturelands and croplands, along with the interwoven wildlife habitats that persist within them (5)—which cover over half of the global land surface (6). However, the capacity for human-modified landscapes to conserve biodiversity depends on the amount and quality of wildlife habitat available (7, 8), the characteristics of the intervening matrix (9), and species’ habitat specialization (10).

The species–area relationship (SAR), which describes the number of species as a function of habitat area, is fundamental to the conservation and management of fragmented habitats (11–13). It has been widely applied to predict species extirpation (i.e., local extinction) rates due to habitat loss (14) and is a cornerstone of protected area design (15–17). The SAR also provides a framework to quantify the biodiversity importance of small habitat remnants (18) and how the intervening matrix may affect species richness in adjacent wildlife habitats (19, 20). This is because the SAR is often best described as a power-law function (21, 22), meaning that expanding small habitat areas accumulates species at a greater rate than larger ones, all else being equal.

Comparisons of SARs between naturally formed islands (e.g., oceanic islands) and anthropogenic habitat remnants (e.g., forest fragments) suggest that a less inhospitable matrix contributes to a decrease in SAR slopes (21, 23) (i.e., the rate at which species richness increases with area) and an increase in SAR intercepts (i.e., the species richness in a 1-ha forest remnant area in log–log space) (21). For example, a multitaxa comparison of 125 SARs from oceanic islands and 135 SARs from terrestrial-matrix habitat remnants found 118% higher SAR intercepts and 37% lower SAR slopes in the latter, based on median values (21). However, oceanic islands are often millions of years old (24) and may not be appropriate for direct comparisons with contemporary terrestrial-matrix habitat remnants. Moreover, the biota in habitat remnants initially consists of relict species (present before fragmentation) and is later supplemented by species from the surrounding landscape, offering a distinct contrast to oceanic islands, which start with no species and are colonized from large landmass sources (25, 26). Recently isolated islands within reservoirs created by river damming represent once-continuous habitat that becomes fragmented into habitat remnants surrounded by an inhospitable water matrix, forming an anthropogenic archipelago. Therefore, reservoir islands serve as clear analogs for understanding the effects of matrix type (aquatic or terrestrial) on SARs for habitat remnants in human-modified landscapes.

**Fig. 1.**
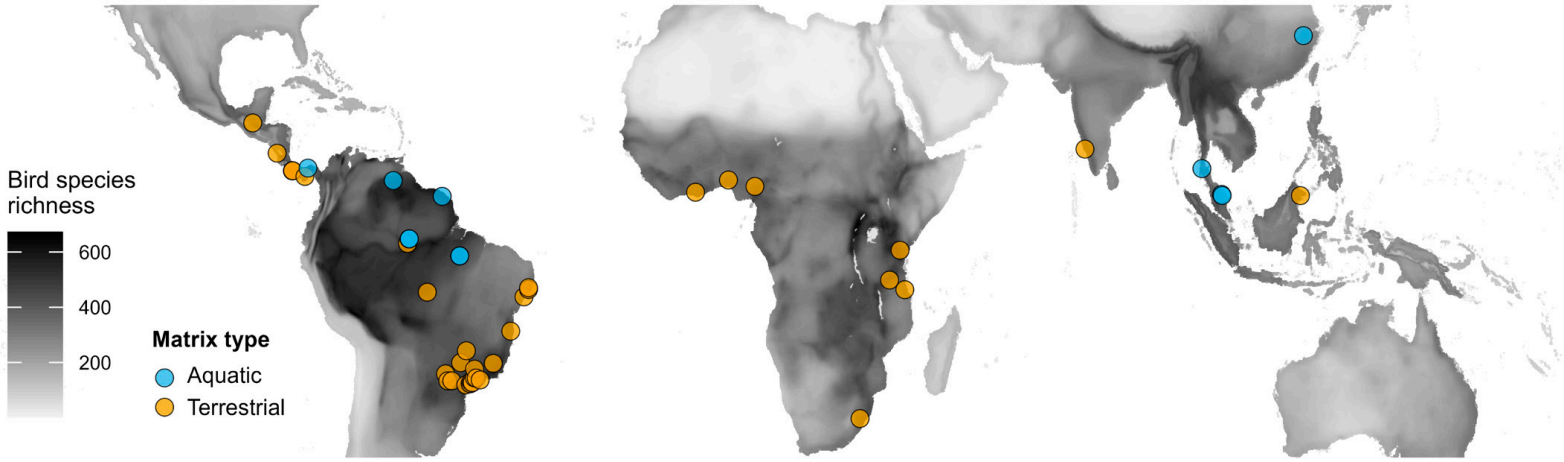
Location of the 50 datasets from tropical and subtropical regions. These datasets were used to examine the effects of the surrounding landscape on avian SARs. Birds were surveyed in forest remnants created by either river damming (aquatic matrix) or clear-cut deforestation (terrestrial matrix). The overall sampling effort included data obtained from 1,005 forest remnants, comprising 336 forest islands and 669 forest fragments. The map was produced using global bird species richness raster data from BiodiversityMapping.org (*Materials and Methods*).

In this study, we quantify the effects of the surrounding landscape on species richness of tropical and subtropical forest remnants embedded within either freshwater aquatic or terrestrial matrices. Herein, the term surrounding landscape comprises both the matrix type (“nonhabitat”) and the amount of tree cover (“habitat”) around forest remnants within a given landscape size. We compare bird species richness for all species and forest-dependent species from 50 datasets comprising either forest islands in water reservoirs or forest fragments in anthropogenic terrestrial matrices (Fig. 1 and *SI Appendix*, Table S1). Applying the SAR for forest remnants of varying size (Type IV curve) (27), we hypothesize that terrestrial matrices are less hostile, more permeable, and provide more resource subsidies for terrestrial birds (28) than an aquatic matrix, resulting in greater species richness in forest fragments than forest islands. Additionally, we predict that the relative benefits of terrestrial matrices will be more substantial in smaller versus larger forest fragments. Specifically, we expect that species richness in small forest remnants (SAR intercept) will be higher and predicted extirpation rates (SAR slope) lower in forest fragments than on similar-sized forest islands, particularly for forest-dependent species.

We also examined two alternative predictions that may account for variation in SAR intercepts and slopes. First, metapopulation dynamics theory predicts that SAR intercepts and slopes are affected by isolation from the source of individuals (e.g., large forest remnants) subsidizing nearby sinks (e.g., small forest remnants) (29, 30). Accordingly, we tested whether isolation from potential sources of individuals affected SAR intercepts and slopes using SAR models that included the effect of the amount of tree cover in the surrounding landscape. This approach reflects isolation as habitat availability within a given landscape size, which has been shown to outperform distance-based metrics in fragmented landscapes (31). Thus, forest remnants surrounded by higher tree cover are considered less isolated and more likely to receive immigrants than those surrounded by lower tree cover (7). Second, extinction debt theory predicts that forest remnants undergo delayed extinctions, which may only be realized many years after isolation (32, 33). Such extinction debt is expected to steepen SAR slopes and reduce intercepts compared to more recently isolated forest remnants. Thus, we additionally modeled the effects of both forest remnant area and time since isolation on species richness.

## Results

**Fig. 2.**
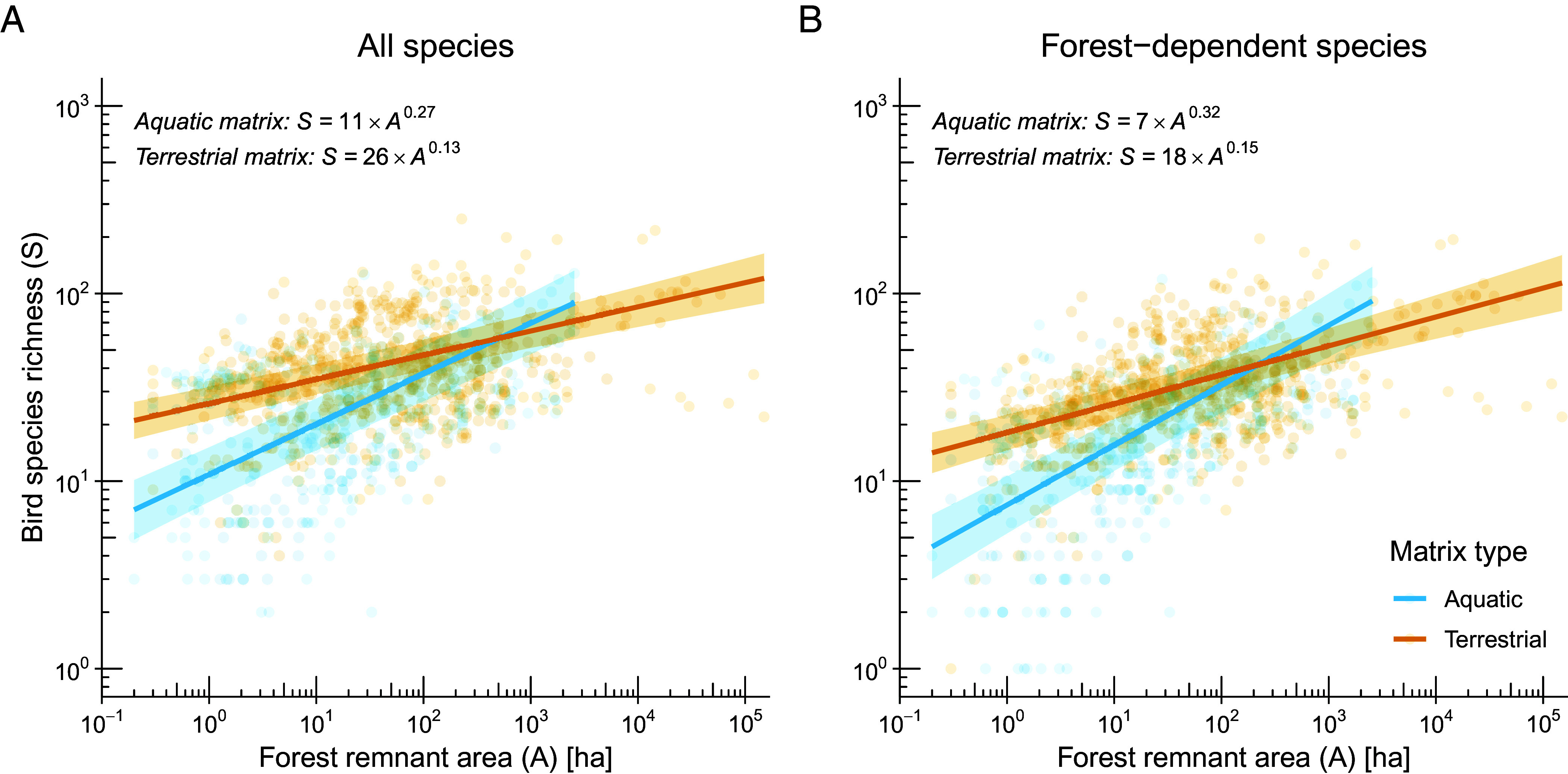
Matrix effect on avian SARs. The Linear Mixed-Effects Models (LMMs) included forest remnants surrounded by either an aquatic or terrestrial matrix based on 50 datasets from tropical and subtropical regions. Compared to forest islands (aquatic matrix), forest fragments (terrestrial matrix) showed higher species richness in a 1-ha forest remnant (i.e., higher regression intercept) and lower predicted extirpation rates (i.e., lower regression slopes), indicating that the terrestrial matrix reduces species’ minimum habitat area requirements and mitigates species loss due to forest loss. This pattern held true for both (*A*) all species and (*B*) forest-dependent species. Data are from 336 forest islands and 669 forest fragments (see *SI Appendix*, Fig. S1 to visualize the distribution of areas of islands and fragments). Blue regression lines are truncated at the size of the largest island. Shaded areas indicate 95% CI. Note that both axes are on a base-10 logarithmic scale, as indicated by the inner tick marks. Regression coefficients were derived from a LMM [log_10_(Bird species richness) ~ log_10_(Forest remnant area) × Matrix type] with dataset identity as a random factor. The estimated parameters displayed in the *Top-Left* corner of panels *A* and *B* are backtransformed to the power-law function.

For all species, the best-fit model in the candidate set included an interactive fixed effect of matrix type on the SAR intercept and slope (*SI Appendix*, Table S2; AICcWt = 0.90). Accordingly, a terrestrial matrix increased the intercept (i.e., more species in 1-ha forest remnants) and decreased the slope (i.e., species richness less sensitive to reductions in forest remnant area). In general, bird species richness within any given 1-ha forest fragment increased by 139% (95% CI: 112 to 157%; from 11 to 26 species), and species loss due to forest shrinkage decreased by 52% (95% CI: 43 to 58%; from 0.27 to 0.13), compared to forest islands (Fig. 2*A* and *SI Appendix*, Table S3). Moreover, the matrix effect was significant for forest-dependent species, with forest fragments showing an intercept 143% higher (95% CI: 111 to 165%; from 7 to 18 species), and a slope 52% lower (95% CI: 43 to 58%; from 0.32 to 0.15) than forest islands (Fig. 2*B* and *SI Appendix*, Table S4). Importantly, our models were fitted primarily to data from relatively small forest remnants (upper quartile = 146.8 ha; n = 1,005; *SI Appendix*, Fig. S1).

While these results allow us to draw inferences about fragmented forest landscapes across the tropics, where forest remnants are typically smaller than 17 ha (34), SAR slopes for larger islands and fragments are more uncertain. For example, it seems unlikely that islands harbor more species than fragments, as predicted for all species beyond 510 ha and for forest-dependent species beyond 216 ha (Fig. 2). Indeed, above the intersection of the SAR regressions up to the largest island (2,551 ha), islands harbored, on average, fewer species than fragments for all species (mean ± SD = 50 ± 34 and 58 ± 42, respectively) and forest-dependent species (38 ± 24 and 45 ± 33). However, these differences were not statistically significant (*t* test: all species, *P* = 0.419, n = 21 islands and 47 fragments; forest-dependent species, *P* = 0.151, n = 40 islands and 124 fragments). Overall, this indicates that, beyond a certain size (e.g., approximately 300 ha), islands and fragments do not differ in their species richness and behave similarly to a continuous habitat (35).

**Fig. 3.**
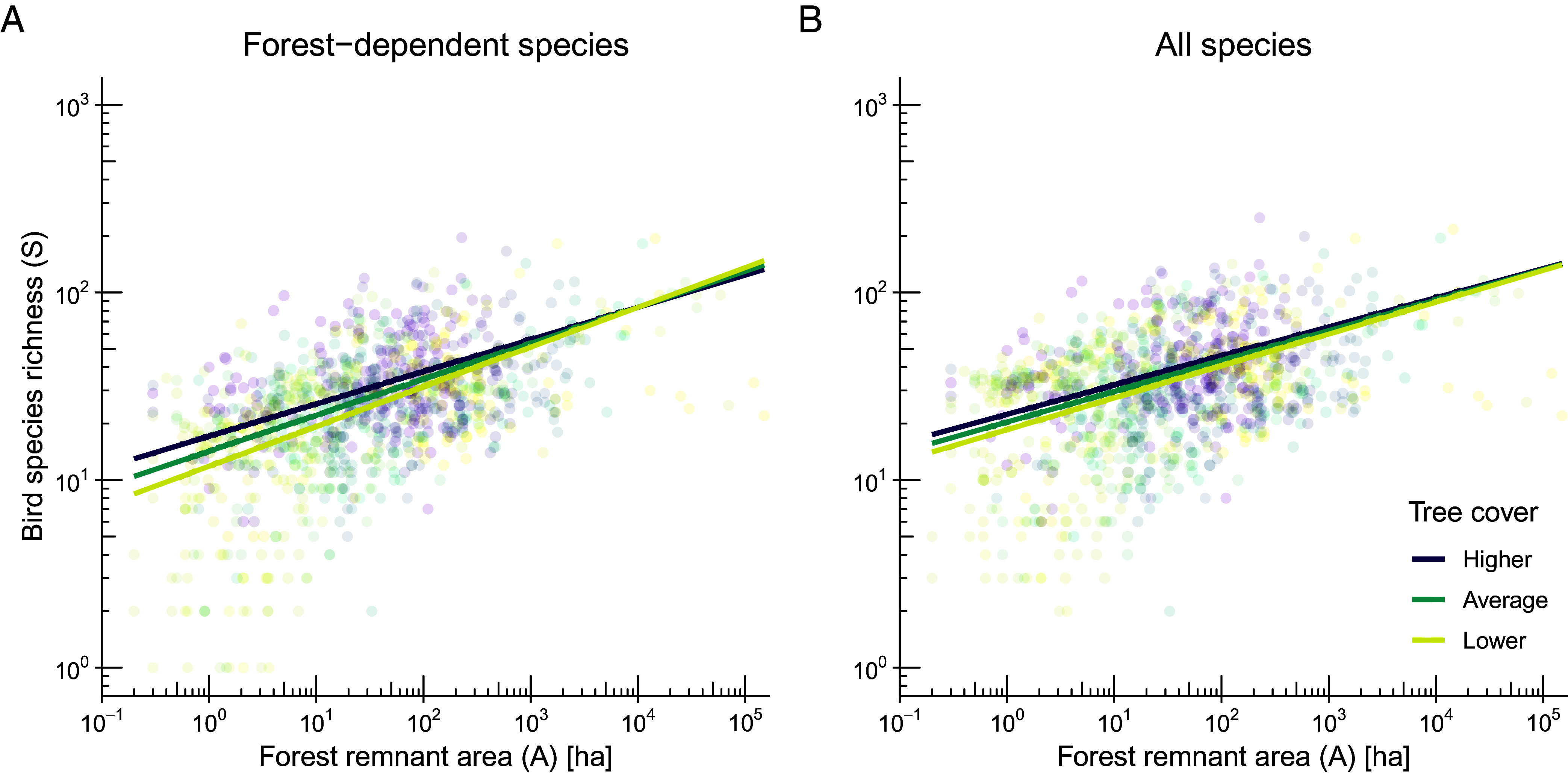
Tree cover effect on avian SARs. The LMMs included forest remnants surrounded by different levels of tree cover within a 300-m buffer around the point location of each of the surveyed forest remnants. Because tree cover and log_10_-forest remnant area are expected to be positively related, we used the residuals of the linear regression model between these two variables as the interactive predictor variable. For simplicity, we refer to these residuals as tree cover, as this approach removes the contribution of forest remnant area to the total tree cover in the local landscape. (*A*) For forest-dependent species, greater amounts of tree cover around forest remnants increased the regression intercept and decreased the slope. (*B*) For all species, only the regression intercept was significantly influenced by tree cover. Data are from 1,005 forest remnants. Regression lines represent the effect of forest remnant area on bird species richness for the mean value of tree cover surrounding forest remnants (average), while lower and higher values correspond to –1 and +1 SD from the mean, respectively. The 95% CI were omitted here to enhance visualization, but they are shown in *SI Appendix*, Fig. S2. Note that both axes are on a base-10 logarithmic scale, as indicated by the inner tick marks. Regression coefficients were derived from a LMM [log_10_(Bird species richness) ~ log_10_(Forest remnant area) × Tree cover] with dataset identity as a random factor.

For forest-dependent species, the best-fit model in the candidate set included an interactive fixed effect of tree cover in the surrounding landscape on the SAR intercept and slope (*SI Appendix*, Table S5; AICcWt = 0.97). Accordingly, as tree cover around forest remnants increased, the intercept increased while the slope decreased (Fig. 3*A* and *SI Appendix*, Fig. S2*A* and Table S6). For all species, tree cover had a significant effect on the SAR intercept but not on the slope (Fig. 3*B* and *SI Appendix*, Fig. S2*B* and Table S7). This indicates that tree cover primarily elevates overall richness rather than altering the rate of species loss as forest remnant area decreases. For example, for a 5-ha forest remnant, one SD of additional tree cover within 300 m increases the expected forest-dependent species richness by 16% (95% CI: 11 to 22%; from 19 to 23 species) and of all species by 9% (95% CI: 4 to 13%; from 26 to 29 species). The same increase in tree cover reduced the SAR slope by 10% (95% CI: 4 to 17%; from 0.19 to 0.17) for forest-dependent species but had no significant effect for all species.

Elevation, latitude, and the size of the regional species pool were all uninformative in explaining the variation in the SAR intercepts and slopes for either all species or forest-dependent species (*SI Appendix*, Tables S2 and S5). Similarly, we found no evidence that isolation time affects the coefficients of avian SARs in a subanalysis of 29 (58%) of the 50 studies for which isolation time is known (*SI Appendix*, Tables S8 and S9).

## Discussion

Avifaunas in forest fragments can benefit from the intervening terrestrial matrix, leading to smaller patch-scale habitat area requirements for species (higher SAR intercept) and a reduced effect of forest remnant area shrinkage on species richness (lower SAR slope) compared to forest islands. Remarkably, the number of species was more than twice as high in small-sized (~1 ha) forest fragments compared to forest islands, reinforcing the notion that terrestrial matrix habitats do contribute to biodiversity persistence in small forest remnants (36). Likewise, the magnitude of species loss with decreasing area was reduced by over 50% in forest fragments relative to forest islands, highlighting the potential of terrestrial matrices to moderate area-driven biodiversity loss. The number of species in forest remnants increased along the gradient from low- to high-quality matrix (9, 37), represented by aquatic and terrestrial matrices, respectively. This pattern is influenced by three core matrix effects associated with species’ dispersal, resource availability, and the abiotic environment (28).

Regarding dispersal through the matrix, species exhibiting more gap-crossing movements also occupy a larger number of forest fragments in southern Amazonia (38). Such movements can be enhanced by the matrix type, as demonstrated in a translocation experiment in Jamaica, where passerine species showed lower movement capacity across a bauxite mining matrix compared to a periurban matrix (39). Moreover, recolonization of previously extirpated species from forest fragments has been documented at the Biological Dynamics of Forest Fragments Project in the Brazilian Amazon as the secondary forest matrix becomes structurally more similar to old-growth forest habitat (40). Collectively, the evidence suggests that more permeable matrices increase species richness in forest remnants by enhancing landscape connectivity, which is undoubtedly greater in terrestrial than in aquatic matrices. Such greater connectivity is expected to lower SAR slopes by reducing extinction risk through the rescue effect, as the demographic and genetic contributions of immigrants of species already present in forest remnants increase their population size and fitness (41). However, the contribution of matrix type to the rescue effect depends on the presence of nearby sources of individuals for forest remnants. For example, in Chile, highly isolated relict forest fragments exhibited higher SAR slopes for birds than land-bridge islands near the mainland (42).

A benign matrix can help maintain or increase species’ population sizes in forest fragments via landscape supplementation or complementation, providing resources similar to or different from those found in forest fragments, respectively (9, 28). This was the case for Amazonian hummingbirds surveyed by mist-netting in forest fragments before and 9 y after isolation (43). Of the three species studied, capture rates in forest fragments remained constant for two species over time but nearly doubled for the third species, despite a decrease in forest area. This outcome likely relates to the ability of the hummingbird species to forage in a variety of habitats, including young second growth (43). As the extirpation risk decreases with increasing population size (26), the ability of species to exploit resources available in the intervening matrix will ensure their persistence in forest fragments, ultimately mitigating species loss over time, as predicted by extinction debt theory (32). Overall, as more species in forest fragments are able supplement or complement their metabolic requirements from resources in the matrix, their minimum forest area requirements will be lower, resulting in higher forest fragment SAR intercepts compared to those of forest islands.

The quality of the matrix also affects the microhabitat structure and abiotic environment in forest fragments, which in turn affects species’ persistence. For instance, among several microclimatic variables examined along the edge to forest interior distance gradient, light intensity was the most important predictor of bird assemblages in Mesoamerican forests (44). Furthermore, population declines of species restricted to shaded environments point to the effects of greater light intensity as a plausible reason for extirpation of light-sensitive species in fragmented tropical forest landscapes (44). Distance to the forest edge also alters vegetation density, canopy cover, and leaf litter depth in forest fragments, thereby degrading the microhabitat structure for forest specialist species, precluding them from persisting in small forest fragments or near forest edges, as shown for terrestrial insectivorous birds in the Brazilian Amazon (45, 46) and Peninsular Malaysia (47). Since the magnitude of edge-associated effects is strongly mediated by the intervening matrix (48), forest fragments within a terrestrial matrix are exposed to weaker edge effects than forest islands within an aquatic matrix (49). Our study extends this finding by examining multiple landscapes across the globe, corroborating that forest fragments retain higher habitat quality than similar-sized forest islands, thereby supporting more bird species.

The matrix effect on both the slope and the intercept of SARs appears to be a general finding (21), with responses varying among different taxonomic groups. For example, a comparison between anthropogenic forest islands and fragments found a positive SAR slope for bats on islands, but a flat slope in fragments (50). Matrix type was also the only factor explaining variance among 28 amphibian SARs from 441 forest remnants surrounded by either an aquatic matrix or five types of terrestrial matrices (20). Despite our coarse matrix classification into either aquatic or terrestrial, SARs derived from avian surveys in Amazonian forest remnants within open matrices—water and pastureland—yielded a much steeper SAR slope for forest islands (*z* = 0.316) (51) than for forest fragments (*z* = 0.191) (52). However, we acknowledge that a more refined assessment of terrestrial matrix quality—e.g., covering the spectrum from bare ground to old secondary forest—is essential to determine the degree to which any terrestrial matrix shapes the SARs of forest fragments.

Beyond matrix effects on the SAR in patchy ecosystems (25), the effect of habitat shrinkage on species loss is determined by the habitat specialization of the focal species group. Indeed, depending on species’ habitat specialization, a SAR may have a positive, flat, or negative slope (53). Therefore, the effect of forest remnant area on species richness will be underestimated if the loss of forest-dependent species is counterbalanced by any gain of non-forest-dependent species, particularly in forest fragments due to the influx of matrix-derived species (25, 54), which stresses the need to assess habitat specialist species separately (55). Likewise, forest-dependent species are more severely affected by forest shrinkage than the overall avifauna, and this effect is more pronounced in forest remnants surrounded by an aquatic than a terrestrial matrix. This suggests that forest-dependent species, which are typically of greatest conservation concern, will benefit the most from matrix enhancement in fragmented forest landscapes.

The amount of tree cover around forest remnants was an important factor affecting the SAR, particularly for forest-dependent species. The SAR for forest-dependent species showed that forest remnants in landscapes with greater tree cover support more species in 1-ha forest remnants (higher intercept) and reduce extirpation rates (lower slope), while only a higher intercept was observed for all species. The limited size of the local landscape (i.e., 300-m buffer) suggests that the influence of tree cover on avian SARs is largely restricted to the immediate vicinity of focal forest remnants. This local landscape may be even smaller if species’ vagility in the landscape is reduced due to dispersal limitation and low matrix permeability to movements (56).

The steeper slope of SARs on oceanic islands compared to continuous habitats (57) implies the operation of not only the sample area effect (18) but also the island effect, with greater extirpation rates on smaller islands due to small population sizes and colonization constraints on more isolated islands (26). However, according to the Habitat Amount Hypothesis (7), habitat remnants in fragmented landscapes are not discrete spatial units. As a result, species richness would be primarily influenced by the amount of habitat in the landscape rather than by habitat patch size (7). Arguably, forest remnants are neither as discrete as expected under the Theory of Island Biogeography nor as continuous as expected under the Habitat Amount Hypothesis (56). However, the effect of tree cover on the SAR suggests that species living in forest remnants may exploit resources available in the surrounding forest habitat (increasing the SAR intercept) and that surrounding forest habitat may serve as a source of immigrants (decreasing the SAR slope), thereby supporting metapopulation dynamics theory. In other words, the more forest habitat available in the local landscape, the lower the influence of forest remnant area on species richness.

Isolation time, elevation, latitude, and the size of the regional species pool had no appreciable effects on the SAR parameters, although this does not imply that they are unimportant. Species extirpations are expected to accumulate over time following habitat shrinkage, thereby steepening the SAR slope (33). However, this trend can be reversed by species turnover, where habitat specialists are replaced by habitat generalists, and by recolonization events by previously extirpated species (18, 40, 54). The importance of extinction debt, which may take place over decades or centuries, still requires further attention, with larger habitat remnants remaining in “debt” over longer timescales compared to smaller ones (32). Forest remnants of known age in our dataset spanned 1 to 200 y since isolation, with most (83%) younger than 50 y (*SI Appendix*, Fig. S3), perhaps precluding statistical resolution of the effect of isolation time on the SAR slope. Similarly, elevation and latitude, both associated with a decrease in species richness (57, 58), also spanned only a short range of variation (59). Additionally, most reservoirs were located at low latitudes and always at low elevations, partly confounding the effects of matrix type and landscape geography. The SAR intercept was also unaffected by the regional bird species pool, supporting the notion that surrounding landscape effects are consistent across different biogeographic realms.

In sum, we provide empirical evidence that matrix type and the amount of tree cover around forest remnants affect the SAR, demonstrating that high-quality surrounding landscapes boost species richness while mitigating species extirpation, particularly for forest-dependent species in small forest fragments. This finding further corroborates that small forest fragments can play a critical role in biodiversity conservation and meeting sustainability goals (60, 61). Moreover, beyond protecting forest remnants themselves, area-based conservation efforts would be greatly enhanced by improving matrix quality (62) and expanding tree cover (63) in otherwise hostile surrounding landscapes. Aligned with this, a global synthesis demonstrated that matrix condition strongly modulates biodiversity outcomes and may even outweigh the effects of habitat loss and fragmentation (64). Finer-scale comparisons are needed to understand how and which resources in the matrix affect and support biodiversity in forest remnants in the long term, especially in the context of different land uses and restoration pathways (3, 65). Conceptualizing human-modified landscapes as potentially benign (5, 66, 67), rather than inhospitable, may reconcile biodiversity conservation with the imperative of agricultural and grazing lands that dominate the Anthropocene.

## Materials and Methods

### Data Collation.

We collated data from 45 studies (51, 52, 63, 68–109), resulting in 50 datasets that featured landscapes created by either river damming (n = 12) or clear-cut deforestation (n = 38) (Fig. 1 and *SI Appendix*, Table S1). Bird surveys were conducted using a variety of methods, including point counts, transect surveys, walkabout surveys, mist-netting, and passive acoustic monitoring. In total, 1,954 bird species were detected through 39,197 incidence records from 336 forest islands and 669 forest fragments, including five Critically Endangered, 12 Endangered, 44 Vulnerable, 83 Near Threatened, and 1,810 Least Concern species, according to the IUCN Red List of Threatened Species (110). The selection criteria for the datasets included a minimum of five fragments or islands located in tropical or subtropical regions (*SI Appendix*, Table S1).

To examine how forest-dependent species affect species richness trends in forest remnants, and whether non-forest-dependent species reduce the slope of the SAR through colonization from the terrestrial matrix (i.e., mass effects) (111), we examined trends for either forest-dependent species or all species. Forest dependency was attributed based on BirdLife International (https://datazone.birdlife.org/home, Accessed on 25 July 2024). The “forest-dependent” subgroup (76.5% of all species) included species with either “high” (496 species) or “medium” (999 species) forest dependency. Although 48 species could be classified as water birds, as they are assigned to the trophic niches “Aquatic predator” or “Herbivore aquatic” (112), their exclusion from the analysis did not change the results, so we present the results including them.

The effects of the surrounding landscape on SARs were quantified by considering the matrix type and the amount of tree cover around forest remnants. The matrix type was classified as either aquatic or terrestrial, with the terrestrial matrix assumed to be less hostile regarding environmental conditions, more prone to species dispersal between forest remnants, and more likely to provide resources for terrestrial birds than the aquatic matrix (28, 50). The amount of tree cover around forest remnants was calculated using 30-m-resolution global maps of forest extent and change from 2000 through 2023, version 1.11 (113). Each pixel value represents the percentage of canopy closure for all vegetation taller than 5 m in height for the year 2000, which was classified as either forest (≥50% tree cover) or nonforest (114). This binary classification is used loosely, as tree cover can comprise both natural forests and tree plantations (e.g., monocultures of oil palm, rubber, pine, or eucalypt) (115). However, tree cover mostly represented natural forests, and tree plantations can be a high-quality matrix for forest species in certain contexts (116), given the structural similarity between the habitat (forest) and the matrix (tree plantation) (117). When bird surveys were conducted before 2002, we used the tree cover map for the year 2000. For surveys conducted from 2002 to 2017 (the year of the most recent bird survey in our database), we updated the tree cover map by subtracting the cumulative tree cover lost from 2001 up to the year prior to the first year of the field survey (118). Tree cover gain was not considered, as the year in which gains occurred is unavailable.

The calculation of the amount of tree cover around forest fragments (hereafter, tree cover) involved several steps. First, since tree cover is a landscape-scale variable, it was necessary to determine the most appropriate landscape scale for measurement (i.e., the local landscape) for the predictive models (119). Hence, we calculated tree cover within 40 different buffer sizes around the point location of each surveyed forest remnant, ranging from 50 to 2,000 m at 50‐m intervals (56). Second, because tree cover and log_10_-forest remnant area are expected to be positively related, we used the residuals of the linear regression model between these two variables across the 40 buffer sizes. This approach removes the contribution of forest remnant area to the total tree cover in the local landscape. Third, for each buffer size, we fitted LMMs using i) species richness for all species and forest-dependent species separately as the response variables, ii) the residuals of tree cover relative to forest remnant area as the predictor variable, and iii) the dataset identity as a random factor, allowing the intercept and the slope to vary across datasets. Next, models were ranked using Akaike’s Information Criterion corrected for small sample sizes (AICc) (120). The size of the local landscape was determined based on the model with the lowest AICc value, which corresponded to the model with the predictor variable measured in a 300-m buffer for all species and forest-dependent species (*SI Appendix*, Fig. S4). Finally, we used the residuals of the relationship between tree cover within the 300-m buffer and log_10_-forest remnant area as our measure of tree cover, since the residuals indicate the amount of tree cover independent of forest remnant area. Accordingly, positive values indicate that the observed tree cover exceeds the predicted values for the corresponding forest remnant area, whereas negative values indicate that it falls below the predicted values.

We defined isolation time for each forest remnant as the year the first sampling took place minus the year forest remnants were created or the approximate year deforestation occurred in the region where each source study was conducted. We obtained isolation time for all 336 forest islands from the 12 datasets, and 282 forest fragments from 17 datasets using information from the source studies, additional literature (121) and discussions with coauthors.

We also assessed whether SARs were affected by variables commonly used to explain biogeographic patterns: elevation (meters above sea level), latitude (absolute distance in decimal degrees from the Equator), and the size of the regional species pool of each study region. Values for these three variables were obtained for the geographic coordinates of the point location of each forest remnant (n = 1,005). Elevation was extracted from the Copernicus Digital Surface Model at a 30-m resolution (GLO-30), version 2023-1. The regional species pool was extracted from BiodiversityMapping.org (122), representing the total number of bird species (excluding seabirds) potentially occurring in each 10 km × 10 km grid cell after spatially overlapping species range maps from BirdLife International, version 7.0 (Fig. 1).

### Statistical Analysis.

To test our predictions, we applied the SAR theoretical framework to bird data to estimate SAR intercepts and slopes based on the linearized power relationship:

$$\log_{10}\left(S\right)=\log_{10}\left(c\right)+z\log_{10}\left(A\right),$$

where *S* = species richness, *A* = forest remnant area in hectares, log_10_(*c*) = intercept, and *z* = slope. Because the SAR slope describes the rate of species loss caused by the reduction in forest remnant area, we estimated matrix effects on this rate by comparing the SAR slopes of forest fragments to those of forest islands. Similarly, we further examined matrix effects by testing whether forest fragments had higher SAR intercepts than forest islands. Since species richness and the number of individuals in a habitat area are positively related (123–125), a higher SAR intercept could imply that species may momentarily spillover into the adjacent terrestrial matrix—but not into the adjacent aquatic matrix—to supplement or complement their minimum habitat area requirements, thereby leading to more individuals and, thus, more species per unit area.

We fitted LMMs to quantify the effects of the surrounding landscape (matrix type and tree cover around forest remnants) and other predictor variables (isolation time, latitude, elevation, and regional species pool) on the slope (i.e., the rate at which species richness increases with area) and intercept of SARs (i.e., the species richness in a 1-ha forest remnant area in log–log space). LMMs were built separately for all species and forest-dependent species, using 50 datasets (excluding isolation time) and 29 datasets (including isolation time). For each fixed effect, three model structures were compared: i) an interactive fixed effect on the SAR, ii) an additive fixed effect on the SAR, and iii) a SAR without any other fixed effects beyond forest remnant area. To account for variation in avian sampling techniques and regional idiosyncrasies among studies, we included dataset identity as a random factor in all SAR models, allowing the intercept and the slope to vary across datasets.

To select among the set of competing candidate models, we used an information-theoretic approach (120). Models were ranked using Akaike’s Information Criterion corrected for small sample sizes (AICc), which favors model fit and simplicity based on the principle of parsimony (avoid adding parameters unless they improve model performance). Lower AICc values indicate a better model fit relative to alternative models in the same candidate set. Next, we calculated an Akaike weight (AICcWt) for each model in the candidate set based on AICc. The designation of the best-fit model was determined by AICcWt ≥ 0.50. Finally, we assessed the robustness of the selected models using a leave-one-out sensitivity analysis, which showed that excluding any single dataset did not alter the direction or significance of the effects.

## Supplementary Material

Appendix 01 (PDF)

Dataset S01 (CSV)

Dataset S02 (CSV)

Dataset S03 (TXT)

## Data Availability

Raw data and the R code used in the analysis are included in the supporting information.
