# Supplementary material for: High-quality surrounding landscapes mitigate avian extirpations from forest remnants
Source: Proc Natl Acad Sci U S A. 2026 Apr 1;123(14):e2521783123. doi: 10.1073/pnas.2521783123 (PMC13056101; doi:10.1073/pnas.2521783123)
Supplement: Supplementary file 1 — Appendix 01 (PDF) [file pnas.2521783123.sapp.pdf]

Supporting Information for

High-quality surrounding landscapes mitigate avian extirpations from forest remnants

Anderson S. Bueno, Chase D. Mendenhall et al.

Anderson S. Bueno
E-mail: anderson.bueno@iffar.edu.br

This PDF file includes:

Figs. S1 to S4
Tables S1 to S9
Legends for Dataset S1 to S2
SI References

Other supporting materials for this manuscript include the following:

Datasets S1 to S2

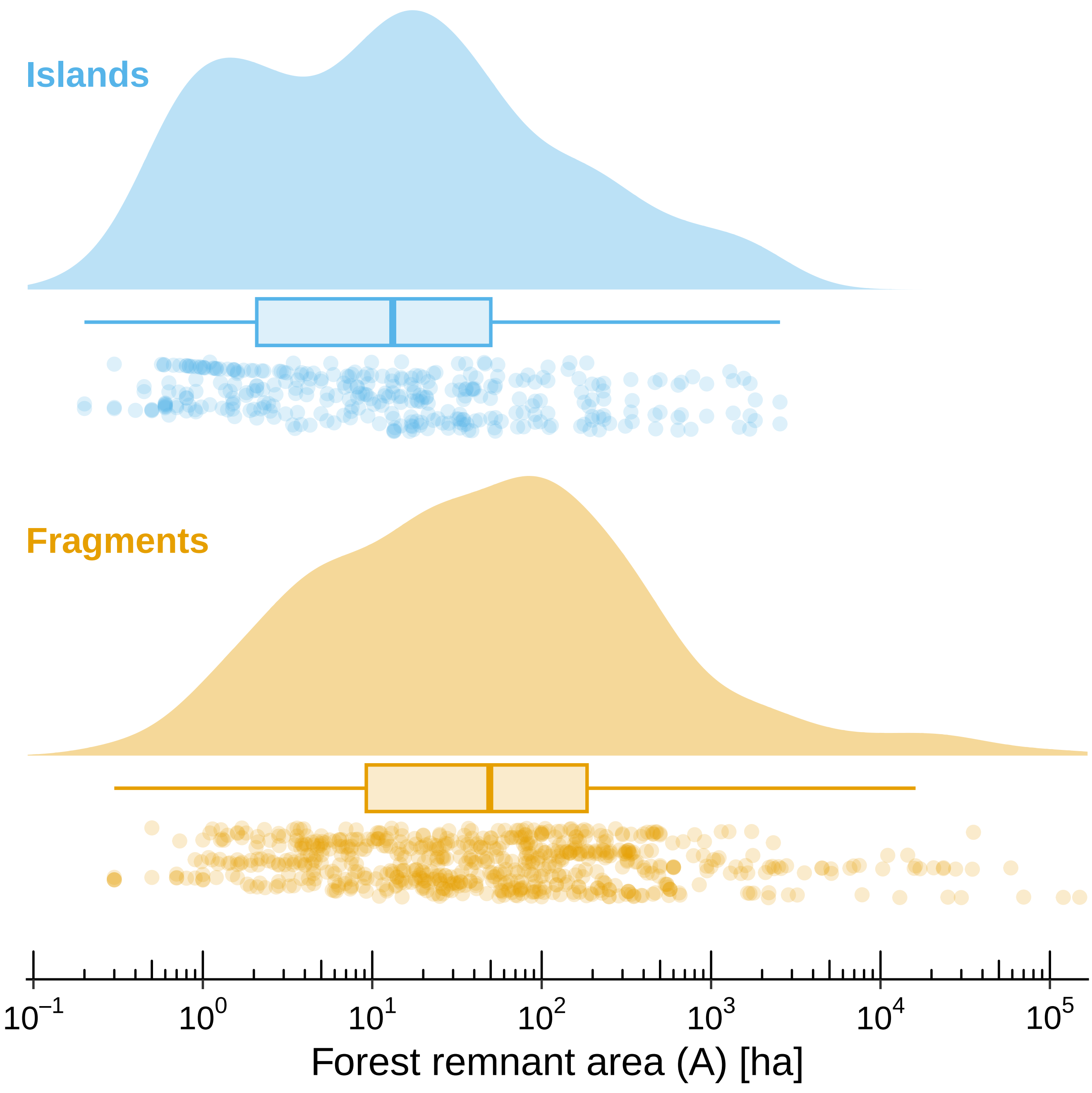

Fig. S1. Raincloud plot showing the area (hectares) of forest remnants, combining a density plot, boxplot, and data points. The information is available for all 1,005 forest remnants from the 50 datasets, including 336 islands and 669 fragments. Area ranges from 0.2 to 2,551 hectares for islands (mean = 123.1, SD = 350.2; median = 13.2, lower–upper quartiles = 2.1–50.1) and from 0.3 to 150,000 hectares for fragments (mean = 1,325.5, SD = 8,842.6; median = 49.4, lower–upper quartiles = 9.2–185.3). Note that the x-axis is on a base-10 logarithmic scale, as indicated by the inner tick marks.

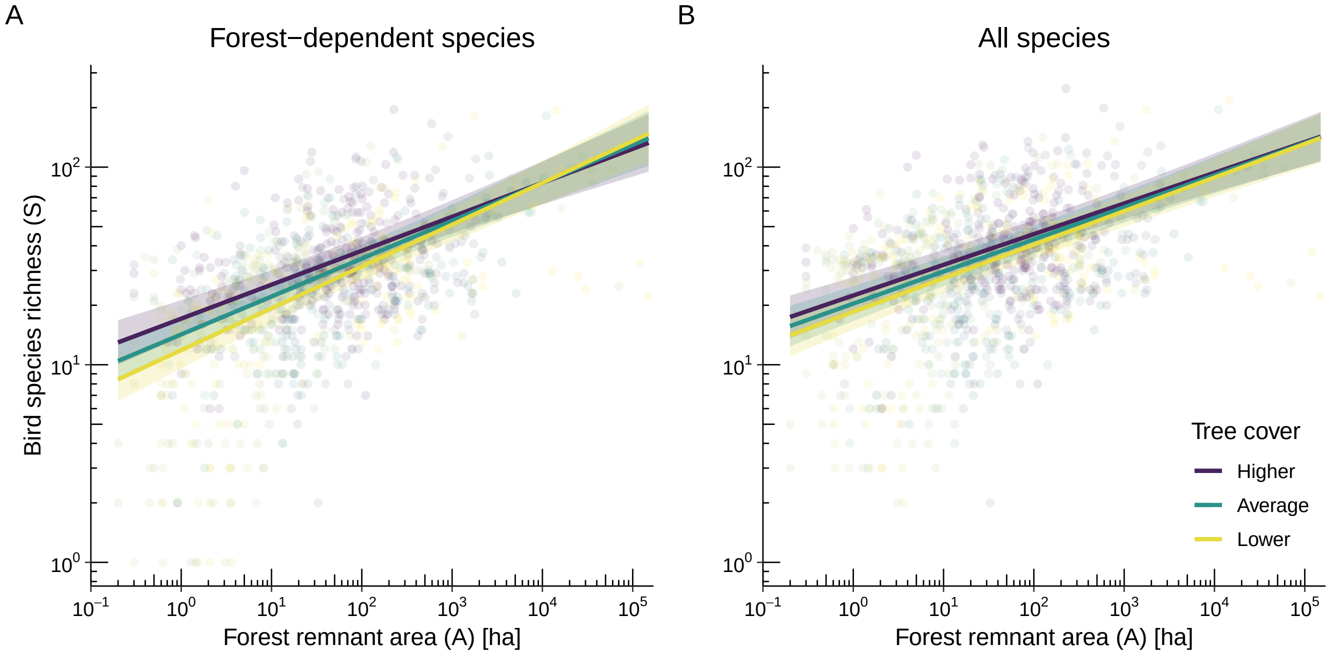

Fig. S2. Tree cover effect on avian species-area relationships. The Linear Mixed-Effects Models included forest remnants surrounded by different levels of tree cover within a 300-m buffer around the point location of each of the surveyed forest remnants. Because tree cover and $\log_{10}$-forest remnant area are expected to be positively related, we used the residuals of the linear regression model between these two variables as the interactive predictor variable, which are referred to as tree cover for simplicity. (*A*) For forest-dependent species, greater amounts of tree cover around forest remnants increased the regression intercept and decreased the slope. (*B*) For all species, only the regression intercept was significantly influenced by tree cover. Data are from 1,005 forest remnants. Regression lines represent the effect of forest remnant area on bird species richness for the mean value of tree cover surrounding forest remnants (average), while lower and higher values correspond to –1 and +1 standard deviations from the mean, respectively. Shaded areas indicate 95% confidence intervals. Note that both axes are on a base-10 logarithmic scale, as indicated by the inner tick marks. Regression coefficients were derived from a Linear Mixed-Effects Model [$\log_{10}$(Bird species richness) ~ $\log_{10}$(Forest remnant area) × Tree cover] with dataset identity as a random factor. This figure differs from Fig. 3 as it shows the confidence intervals and uses a shorter y-axis range.

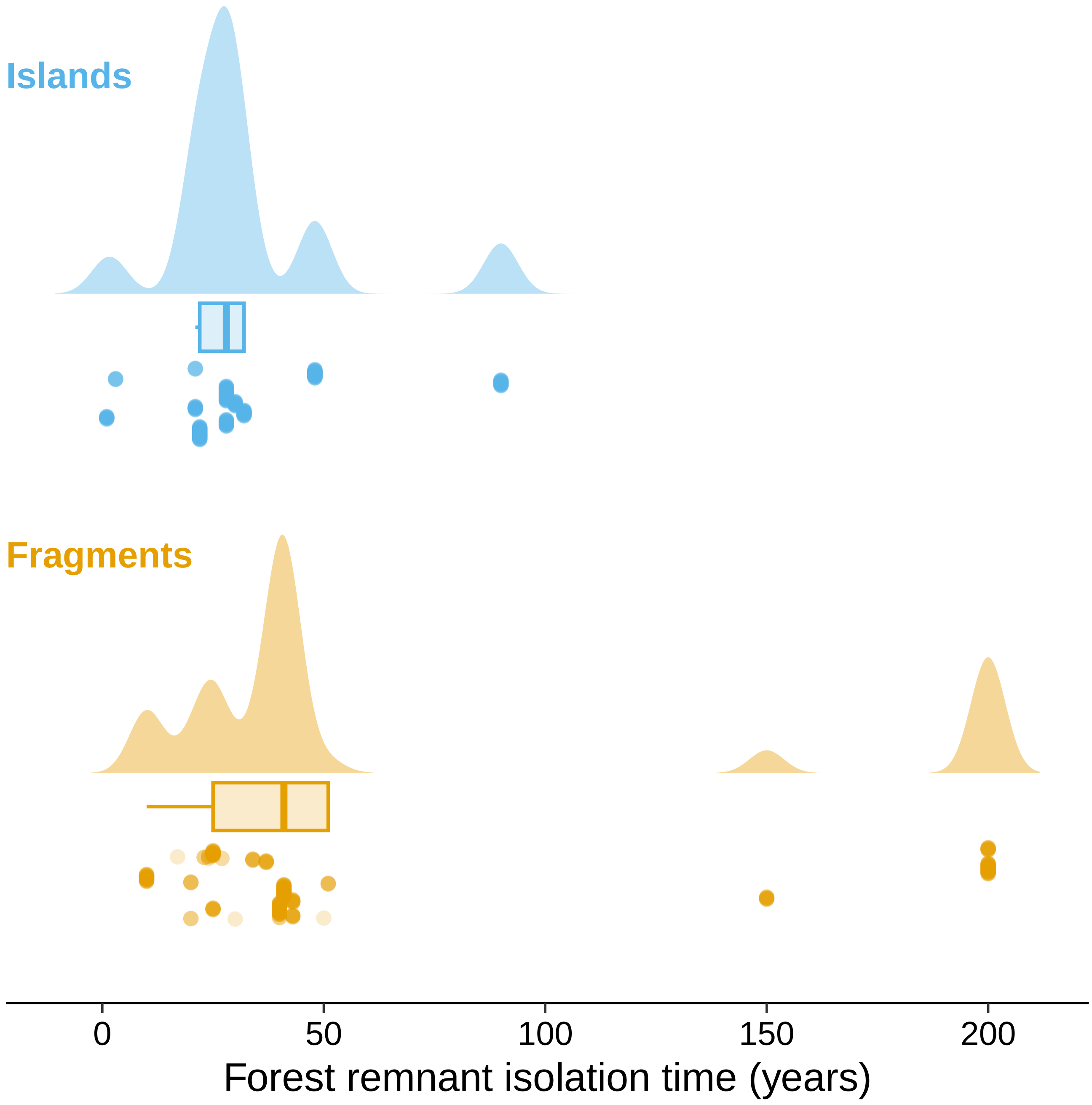

Fig. S3. Raincloud plot showing the isolation time (years) of forest remnants, combining a density plot, boxplot, and data points. The information is available for 618 forest remnants from 29 datasets, including 336 islands and 282 fragments. There is substantial point overlap because the same isolation time is typically assigned to all forest remnants within each dataset. Isolation time ranges from 1 to 90 years for islands (mean = 33.0, SD = 20.4; median = 28, lower–upper quartiles = 22–32) and from 10 to 200 years for fragments (mean = 70.4, SD = 69.3; median = 41, lower–upper quartiles = 25–51).

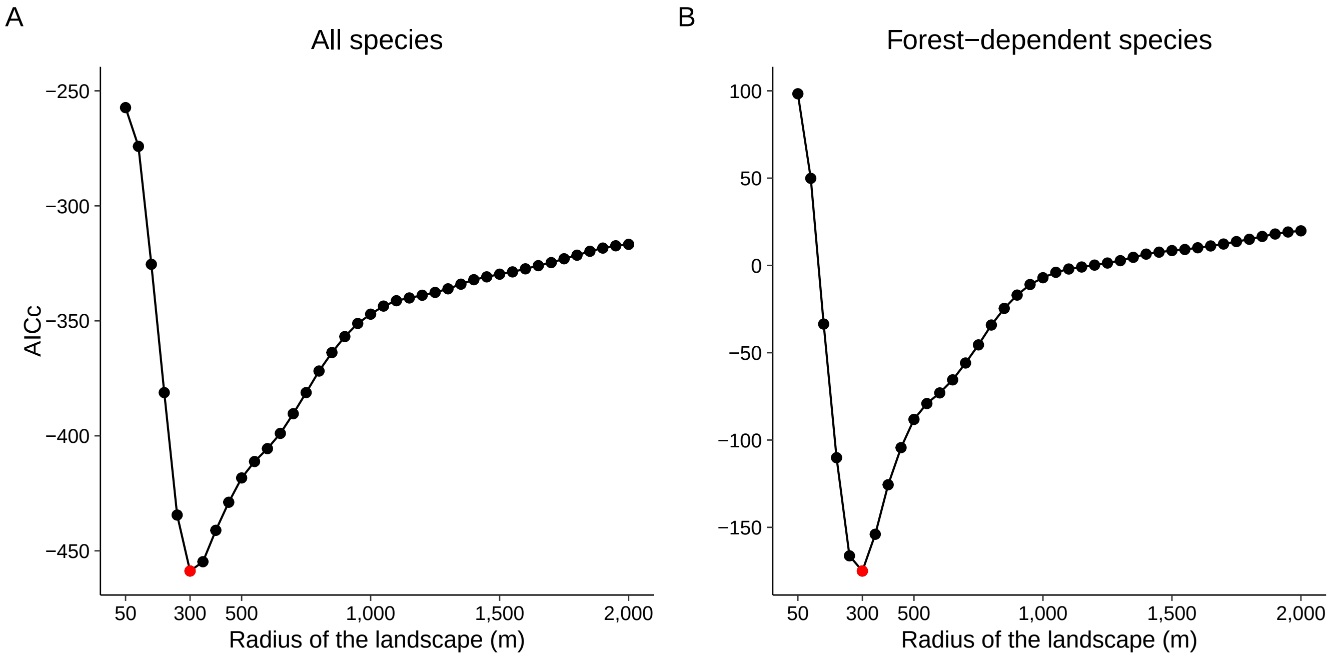

Fig. S4. Multi-scale analysis employed to determine the 'scale of effect' – the landscape scale at which the relationship between bird species richness and tree cover peaks – for (*A*) all species and (*B*) forest-dependent species. Tree cover was calculated within 40 different buffer sizes around the point location of each of the 1,005 surveyed forest remnants from 50 datasets, ranging from 50 to 2,000 m at 50-m intervals. Because tree cover and $\log_{10}$-forest remnant area are expected to be positively correlated, we used the residuals of the linear regression model between these two variables across the 40 buffer sizes as the predictor variable. Next, for each buffer size, we fitted Linear Mixed-Effects Models (LMMs) using (i) species richness for all species and forest-dependent species separately as the response variables, (ii) the residuals of tree cover relative to forest remnant area as the predictor variable, and (iii) the dataset identity as a random factor, allowing slopes to vary across datasets. Finally, the models were ranked using Akaike's Information Criterion corrected for small sample sizes (AICc). The 'scale of effect' was determined based on the model with the lowest AICc value, which corresponded to the model with the predictor variable measured at a 300-m buffer size (i.e., the local landscape, indicated by the red point) for all species and forest-dependent species.

Table S1. Description of the datasets used in this study, including the mean geographic coordinates of the forest remnants, matrix type, number of forest remnants (either forest fragments or forest islands), range of forest remnant area in hectares (ha), approximate mean isolation time of the forest remnants in years (yr), and the total number of species recorded

datasetID (reference number)	Latitude	Longitude	Matrix type	Forest remnants	Min. size (ha)	Max. size (ha)	Isolation time (yr)	Total species
Anciaes2000 (1)	-22.9922	-46.6449	terrestrial	12	15.0	150,000.0	28	100
Anjos2004 (2)	-23.4434	-51.1592	terrestrial	14	11.0	656.0	42	175
Araujo2013 (3)	-9.1639	-35.8243	terrestrial	15	22.9	7,787.5	–	130
Aurelio-Silva2016 (4)	-1.6636	-59.6823	aquatic	27	3.5	1,690.0	22	53
Bispo2010 (5)	-20.6638	-49.3903	terrestrial	17	58.6	2,192.8	–	106
Boesing2018MG (6)	-21.6495	-46.6090	terrestrial	40	2.3	571.9	–	71
Boesing2018SP (6)	-23.0273	-46.2943	terrestrial	52	1.8	851.9	–	85
Bueno2018 (7)	-4.2751	-49.5673	aquatic	36	3.4	2,551.4	22	206
Bueno2020 (8)	-1.6697	-59.6740	aquatic	33	0.6	1,698.8	28	109
Carrara2015 (9)	16.1321	-90.8956	terrestrial	17	2.8	91.9	40	73
Claessens2002 (10)	4.8633	-53.0711	aquatic	15	0.3	28.0	1	148
Daily2001 (11)	8.7882	-82.9847	terrestrial	9	0.3	227.0	25	257
Dami2013 (12)	6.3861	9.3736	terrestrial	20	0.3	23.3	40	121
Develey2004CAU (13)	-23.7223	-47.0836	terrestrial	8	14.0	175.0	–	83
Develey2004IBI (13)	-23.6076	-47.3123	terrestrial	8	24.0	491.0	–	72
Develey2004TAP (13)	-23.8879	-47.4639	terrestrial	8	19.0	158.0	–	108
Edwards2010 (14)	4.9729	118.3043	terrestrial	12	0.7	87.1	43	78
Feeley2003 (15)	7.2845	-62.8428	aquatic	29	0.2	189.8	32	87
Gillespie2001 (16)	11.4879	-86.0308	terrestrial	7	420.0	5,132.0	–	69
Hatfield2020 (17)	-23.3141	-45.4884	terrestrial	34	4.5	2,094.7	–	172
Henriques2021 (18)	-4.2712	-49.5715	aquatic	18	5.4	2,551.4	21	121
Holbech2005 (19)	5.4946	-2.4821	terrestrial	15	2,360.0	58,790.0	–	146
Irving2018 (20)	9.1181	98.6298	aquatic	23	0.8	49.8	30	72
Jensen2005 (21)	-9.4653	39.3170	terrestrial	11	300.0	2,500.0	–	100
Jensen2020 (22)	-7.9830	36.2683	terrestrial	11	110.0	16,110.0	150	71
Jyothi2015 (23)	12.1155	75.2752	terrestrial	15	1.6	24.4	–	107
Kormann2018 (24)	8.7813	-82.9649	terrestrial	49	0.9	1,089.0	41	209
Krugel2000 (25)	-23.4473	-51.9615	terrestrial	5	7.6	59.0	51	143
Lasky2010 (26)	7.9191	-80.4849	terrestrial	5	80.0	1,115.0	20	71
Lees2008 (27)	-9.8505	-56.0500	terrestrial	30	1.1	14,476.1	10	326
Luypaert2022 (28)	-1.6576	-59.6423	aquatic	71	0.4	1,698.8	28	76
Manu2007 (29)	7.3713	4.0911	terrestrial	44	14.0	445.0	200	154
Marini2001 (30)	-18.8417	-48.2444	terrestrial	6	7.5	230.0	–	143
Martensen2012CAU (31)	-23.7237	-47.0751	terrestrial	17	2.1	158.4	–	62
Martensen2012RG (31)	-24.0746	-48.3457	terrestrial	17	4.8	92.3	–	70
Martensen2012TAP (31)	-23.8784	-47.4616	terrestrial	19	2.8	156.5	–	87
Mendenhall2016 (32)	8.7979	-82.9421	terrestrial	9	4.0	593.0	37	229
Moore2008 (33)	9.1970	-79.8693	aquatic	29	1.6	1,560.0	90	143
Morante-Filho2015 (34)	-15.8303	-39.3780	terrestrial	16	2.1	2,330.8	–	150
Nasruddin-Roshidi2021 (35)	5.1654	102.5591	aquatic	7	1.6	23.8	3	100
Oliveira2020 (36)	-10.5640	-36.7394	terrestrial	10	0.7	241.2	–	92
Ribon2003 (37)	-20.7709	-42.8718	terrestrial	41	1.0	384.5	–	169
Ruiz-Gutierrez2010 (38)	8.7784	-82.9464	terrestrial	7	1.4	224.0	34	171
Silveira2003 (39)	-9.4311	-35.8311	terrestrial	15	25.0	800.0	–	208
Stouffer2011 (40)	-2.3654	-59.9827	terrestrial	11	1.1	101.2	24	117
Uezu2011 (41)	-22.3794	-52.3968	terrestrial	22	28.3	35,396.1	25	98
Ulrich2016 (42)	-3.4076	38.3602	terrestrial	12	1.1	178.8	200	69
Wang2012 (43)	29.5318	118.9012	aquatic	42	0.3	1,289.2	48	112
Wethered2005 (44)	-29.2433	30.3618	terrestrial	9	0.5	273.0	–	52
Yong2011 (45)	4.9819	102.6771	aquatic	6	1.1	184.7	21	75

Table S2. Model selection for 50 datasets and all bird species. Ranking of Linear Mixed-Effects Models (LMMs) based on 1,954 bird species (S) and the area (A) of 1,005 forest remnants from tropical and subtropical regions. The models accounted for possible interactive and additive effects on species-area relationships for five fixed factors, namely: (1) $matrixType$ (aquatic or terrestrial); (2) $treeCover300m$ (residuals of the relationship between tree cover within a 300-m buffer landscape size and $\log_{10}$-forest remnant area); (3) $elevation$ (meters above sea level); (4) $latitude$ (absolute distance in decimal degrees from the Equator); and (5) $speciesPool$ (expected number of bird species based on overlapping species range maps from BirdLife International). Models were ranked using Akaike's Information Criterion corrected for small sample sizes (AICc). Lower AICc values indicate better model fit relative to alternative models in the same candidate set. Model comparison variables include: *K* (the number of estimated parameters for each model); *AICc*; $\triangle$*AICc* (the difference in *AICc* relative to the model with the lowest *AICc*); *AICcWt* (the proportion of the total amount of predictive power provided by the full set of models contained in the model being assessed); *Cum.Wt* (the cumulative Akaike weights); and *LL* (the log-likelihood of each model, which indicates how likely the model is, given the data used)

Model: 50 datasets, all species	K	AICc	$\triangle$AICc	AICcWt	Cum.Wt	LL
$\log_{10}(S) = \log_{10}(c) + z\log_{10}(A) \times matrixType$	8	-1017.05	0	0.90	0.90	516.60
$\log_{10}(S) = \log_{10}(c) + z\log_{10}(A) + treeCover300m$	7	-1010.82	6.23	0.04	0.94	512.47
$\log_{10}(S) = \log_{10}(c) + z\log_{10}(A) \times treeCover300m$	8	-1010.27	6.78	0.03	0.97	513.21
$\log_{10}(S) = \log_{10}(c) + z\log_{10}(A) \times latitude$	8	-1010.25	6.80	0.03	1	513.20
$\log_{10}(S) = \log_{10}(c) + z\log_{10}(A) + matrixType$	7	-996.65	20.40	0	1	505.38
$\log_{10}(S) = \log_{10}(c) + z\log_{10}(A)$	6	-994.88	22.17	0	1	503.48
$\log_{10}(S) = \log_{10}(c) + z\log_{10}(A) + speciesPool$	7	-994.67	22.38	0	1	504.39
$\log_{10}(S) = \log_{10}(c) + z\log_{10}(A) + elevation$	7	-993.83	23.22	0	1	503.97
$\log_{10}(S) = \log_{10}(c) + z\log_{10}(A) + latitude$	7	-992.87	24.18	0	1	503.49
$\log_{10}(S) = \log_{10}(c) + z\log_{10}(A) \times speciesPool$	8	-992.64	24.41	0	1	504.39
$\log_{10}(S) = \log_{10}(c) + z\log_{10}(A) \times elevation$	8	-991.98	25.07	0	1	504.06

Table S3. Matrix effect on SAR for 50 datasets and all bird species. Fixed-effect estimates of the best-fit model based on 1,954 bird species from 336 forest islands and 669 forest fragments. The matrix effect was quantified using a Linear Mixed-Effects Model (LMM) framework. In this model, $matrixType$ (aquatic or terrestrial) was included as a fixed interactive effect on the intercept and slope of the relationship between the number of all species and forest remnant area in $\log_{10}$-$\log_{10}$ space (i.e., $\log_{10}(S) = \log_{10}(c) + z\log_{10}(A) \times matrixType$). Intercept and slope values in Fig. 2*A* were calculated as follows. For aquatic matrix: intercept = $10^{1.035}$ = 10.83 $\approx$ 11; slope = 0.269 $\approx$ 0.27. For terrestrial matrix: intercept = $10^{(1.035 + 0.378)}$ = $10^{1.413}$ = 25.89 $\approx$ 26 species; slope = 0.269 - 0.140 = 0.129 $\approx$ 0.13

Fixed effects	Estimate	Std. Error	df	t-value	p-value
Intercept	1.035	0.074	40.687	13.951	< 0.001
$\log_{10}(A)$	0.269	0.023	39.114	11.717	< 0.001
$matrixType$ (terrestrial)	0.378	0.087	43.068	4.365	< 0.001
$\log_{10}(A) : matrixType$ (terrestrial)	-0.140	0.027	43.016	-5.151	< 0.001

Table S4. Matrix effect on SAR for 50 datasets and forest-dependent bird species. Fixed-effect estimates of a model based on 1,495 forest-dependent bird species from 336 forest islands and 669 forest fragments. The matrix effect was quantified using a Linear Mixed-Effects Model (LMM) framework. In this model, $matrixType$ (aquatic or terrestrial) was included as a fixed interactive effect on the intercept and slope of the relationship between the number of forest-dependent species and forest remnant area in $\log_{10}$-$\log_{10}$ space (i.e., $\log_{10}(S) = \log_{10}(c) + z\log_{10}(A) \times matrixType$). Intercept and slope values in Fig. 2*B* were calculated as follows. For aquatic matrix: intercept = $10^{0.873}$ = 7.46 $\approx$ 7; slope = 0.319 $\approx$ 0.32. For terrestrial matrix: intercept = $10^{(0.873 + 0.386)}$ = $10^{1.259}$ = 18.20 $\approx$ 18 species; slope = 0.319 - 0.165 = 0.154 $\approx$ 0.15

Fixed effects	Estimate	Std. Error	df	t-value	p-value
Intercept	0.873	0.077	39.914	11.265	< 0.001
$\log_{10}(A)$	0.319	0.028	39.567	11.433	< 0.001
$matrixType$ (terrestrial)	0.386	0.091	42.605	4.243	< 0.001
$\log_{10}(A) : matrixType$ (terrestrial)	-0.165	0.033	43.122	-5.018	< 0.001

Table S5. Model selection for 50 datasets and forest-dependent bird species. Ranking of Linear Mixed-Effects Models (LMMs) based on 1,495 forest-dependent bird species (S) and the area (A) of 1,005 forest remnants from tropical and subtropical regions. The models accounted for possible interactive and additive effects on species-area relationships for five fixed factors, namely: (1) $matrixType$ (aquatic or terrestrial); (2) $treeCover300m$ (residuals of the relationship between tree cover within a 300-m buffer landscape size and $\log_{10}$-forest remnant area); (3) $elevation$ (meters above sea level); (4) $latitude$ (absolute distance in decimal degrees from the Equator); and (5) $speciesPool$ (expected number of bird species based on overlapping species range maps from BirdLife International). Models were ranked using Akaike's Information Criterion corrected for small sample sizes (AICc). Lower AICc values indicate better model fit relative to alternative models in the same candidate set. Model comparison variables include: *K* (the number of estimated parameters for each model); *AICc*; $\triangle$*AICc* (the difference in *AICc* relative to the model with the lowest *AICc*); *AICcWt* (the proportion of the total amount of predictive power provided by the full set of models contained in the model being assessed); *Cum.Wt* (the cumulative Akaike weights); and *LL* (the log-likelihood of each model, which indicates how likely the model is, given the data used)

Model: 50 datasets, forest-dependent species	K	AICc	ΔAICc	AICcWt	Cum.Wt	LL
$\log_{10}(S) = \log_{10}(c) + z\log_{10}(A) \times treeCover300m$	8	-807.31	0	0.97	0.97	411.73
$\log_{10}(S) = \log_{10}(c) + z\log_{10}(A) + treeCover300m$	7	-800.65	6.67	0.03	1	407.38
$\log_{10}(S) = \log_{10}(c) + z\log_{10}(A) \times matrixType$	8	-788.27	19.05	0	1	402.20
$\log_{10}(S) = \log_{10}(c) + z\log_{10}(A) \times latitude$	8	-783.36	23.95	0	1	399.75
$\log_{10}(S) = \log_{10}(c) + z\log_{10}(A) + speciesPool$	7	-770.45	36.86	0	1	392.28
$\log_{10}(S) = \log_{10}(c) + z\log_{10}(A) + elevation$	7	-769.98	37.33	0	1	392.05
$\log_{10}(S) = \log_{10}(c) + z\log_{10}(A) + matrixType$	7	-769.09	38.22	0	1	391.60
$\log_{10}(S) = \log_{10}(c) + z\log_{10}(A) \times elevation$	8	-768.55	38.77	0	1	392.34
$\log_{10}(S) = \log_{10}(c) + z\log_{10}(A) \times speciesPool$	8	-768.42	38.89	0	1	392.28
$\log_{10}(S) = \log_{10}(c) + z\log_{10}(A)$	6	-768.32	38.99	0	1	390.20
$\log_{10}(S) = \log_{10}(c) + z\log_{10}(A) + latitude$	7	-766.32	41.00	0	1	390.21

Table S6. Tree cover effect on SAR for 50 datasets and forest-dependent bird species. Fixed-effect estimates of the best-fit model based on 1,495 forest-dependent bird species from 336 forest islands and 669 forest fragments. The effect of tree cover around forest remnants was quantified using a Linear Mixed-Effects Model (LMM) framework. In this model, $treeCover300m$ (residuals of the relationship between tree cover within a 300-m buffer landscape size and $\log_{10}$-forest remnant area) was included as a fixed interactive effect on the intercept and slope of the relationship between the number of forest-dependent species and forest remnant area in $\log_{10}$-$\log_{10}$ space (i.e., $\log_{10}(S) = \log_{10}(c) + z\log_{10}(A) \times treeCover300m$)

Fixed effects	Estimate	Std. Error	df	t-value	p-value
Intercept	1.154	0.045	47.745	25.379	< 0.001
$\log_{10}(A)$	0.192	0.018	49.591	10.952	< 0.001
$treeCover300m$	0.003	0.001	908.752	5.564	< 0.001
$\log_{10}(A) : treeCover300m$	-0.001	< 0.001	898.492	-2.965	0.003

Table S7. Tree cover effect on SAR for 50 datasets and all bird species. Fixed-effect estimates of a model based on 1,954 bird species from 336 forest islands and 669 forest fragments. The effect of tree cover around forest remnants was quantified using a Linear Mixed-Effects Model (LMM) framework. In this model, $treeCover300m$ (residuals of the relationship between tree cover within a 300-m buffer landscape size and $\log_{10}$-forest remnant area) was included as a fixed interactive effect on the intercept and slope of the relationship between the number of all species and forest remnant area in $\log_{10}$-$\log_{10}$ space (i.e., $\log_{10}(S) = \log_{10}(c) + z\log_{10}(A) \times treeCover300m$)

Fixed effects	Estimate	Std. Error	df	t-value	p-value
Intercept	1.309	0.045	47.814	28.924	< 0.001
$\log_{10}(A)$	0.163	0.015	48.901	10.982	< 0.001
$treeCover300m$	0.002	< 0.001	894.541	3.214	0.001
$\log_{10}(A) : treeCover300m$	-0.0003	< 0.001	872.573	-1.221	0.222

Table S8. Model selection for 29 datasets comprising forest remnants with known isolation time and all bird species. Ranking of Linear Mixed-Effects Models (LMMs) based on 1,576 bird species (S) and the area (A) of 618 forest remnants from tropical and subtropical regions. The models accounted for possible interactive and additive effects on species-area relationships for six fixed factors, namely: (1) $matrixType$ (aquatic or terrestrial); (2) $treeCover300m$ (residuals of the relationship between tree cover within a 300-m buffer landscape size and $\log_{10}$-forest remnant area); (3) $elevation$ (meters above sea level); (4) $latitude$ (absolute distance in decimal degrees from the Equator); (5) $speciesPool$ (expected number of bird species based on overlapping species range maps from BirdLife International); and (6) $isolationTime$ (the year forest remnants were created or the approximate year deforestation happened in the region where the source study was conducted). Models were ranked using Akaike's Information Criterion corrected for small sample sizes (AICc). Lower AICc values indicate better model fit relative to alternative models in the same candidate set. Model comparison variables include: *K* (the number of estimated parameters for each model); *AICc*; $\triangle$*AICc* (the difference in *AICc* relative to the model with the lowest *AICc*); *AICcWt* (the proportion of the total amount of predictive power provided by the full set of models contained in the model being assessed); *Cum.Wt* (the cumulative Akaike weights); and *LL* (the log-likelihood of each model, which indicates how likely the model is, given the data used)

Model: 29 datasets, all species	K	AICc	$\triangle$AICc	AICcWt	Cum.Wt	LL
$\log_{10}(S) = \log_{10}(c) + z\log_{10}(A) + treeCover300m$	7	-530.68	0	0.67	0.67	272.43
$\log_{10}(S) = \log_{10}(c) + z\log_{10}(A) \times treeCover300m$	8	-528.63	2.05	0.24	0.91	272.43
$\log_{10}(S) = \log_{10}(c) + z\log_{10}(A) \times matrixType$	8	-526.32	4.36	0.08	0.98	271.28
$\log_{10}(S) = \log_{10}(c) + z\log_{10}(A) \times latitude$	8	-522.82	7.86	0.01	0.99	269.53
$\log_{10}(S) = \log_{10}(c) + z\log_{10}(A) + matrixType$	7	-518.38	12.30	0	1	266.28
$\log_{10}(S) = \log_{10}(c) + z\log_{10}(A)$	6	-517.55	13.13	0	1	264.84
$\log_{10}(S) = \log_{10}(c) + z\log_{10}(A) + isolationTime$	7	-516.71	13.97	0	1	265.45
$\log_{10}(S) = \log_{10}(c) + z\log_{10}(A) + speciesPool$	7	-516.54	14.14	0	1	265.36
$\log_{10}(S) = \log_{10}(c) + z\log_{10}(A) \times speciesPool$	8	-516.31	14.38	0	1	266.27
$\log_{10}(S) = \log_{10}(c) + z\log_{10}(A) + elevation$	7	-516.05	14.63	0	1	265.12
$\log_{10}(S) = \log_{10}(c) + z\log_{10}(A) + latitude$	7	-515.74	14.94	0	1	264.96
$\log_{10}(S) = \log_{10}(c) + z\log_{10}(A) \times isolationTime$	8	-515.67	15.02	0	1	265.95
$\log_{10}(S) = \log_{10}(c) + z\log_{10}(A) \times elevation$	8	-514.29	16.39	0	1	265.27

Table S9. Model selection for 29 datasets comprising forest remnants with known isolation time and forest-dependent bird species. Ranking of Linear Mixed-Effects Models (LMMs) based on 1,225 forest-dependent bird species (S) and the area (A) of 618 forest remnants from tropical and subtropical regions. The models accounted for possible interactive and additive effects on species-area relationships for six fixed factors, namely: (1) $matrixType$ (aquatic or terrestrial); (2) $treeCover300m$ (residuals of the relationship between tree cover within a 300-m buffer landscape size and $\log_{10}$-forest remnant area); (3) $elevation$ (meters above sea level); (4) $latitude$ (absolute distance in decimal degrees from the Equator); (5) $speciesPool$ (expected number of bird species based on overlapping species range maps from BirdLife International); and (6) $isolationTime$ (the year forest remnants were created or the approximate year deforestation happened in the region where the source study was conducted). Models were ranked using Akaike's Information Criterion corrected for small sample sizes (AICc). Lower AICc values indicate better model fit relative to alternative models in the same candidate set. Model comparison variables include: *K* (the number of estimated parameters for each model); *AICc*; Δ*AICc* (the difference in *AICc* relative to the model with the lowest *AICc*); *AICcWt* (the proportion of the total amount of predictive power provided by the full set of models contained in the model being assessed); *Cum.Wt* (the cumulative Akaike weights); and *LL* (the log-likelihood of each model, which indicates how likely the model is, given the data used)

Model: 29 datasets, forest-dependent species	K	AICc	ΔAICc	AICcWt	Cum.Wt	LL
$\log_{10}(S) = \log_{10}(c) + z\log_{10}(A) + treeCover300m$	7	-369.51	0	0.52	0.52	191.84
$\log_{10}(S) = \log_{10}(c) + z\log_{10}(A) \times treeCover300m$	8	-369.32	0.19	0.48	1	192.78
$\log_{10}(S) = \log_{10}(c) + z\log_{10}(A) \times matrixType$	8	-351.64	17.86	0	1	183.94
$\log_{10}(S) = \log_{10}(c) + z\log_{10}(A) \times latitude$	8	-348.56	20.95	0	1	182.40
$\log_{10}(S) = \log_{10}(c) + z\log_{10}(A) + isolationTime$	7	-344.95	24.55	0	1	179.57
$\log_{10}(S) = \log_{10}(c) + z\log_{10}(A)$	6	-344.43	25.08	0	1	178.28
$\log_{10}(S) = \log_{10}(c) + z\log_{10}(A) + matrixType$	7	-344.34	25.17	0	1	179.26
$\log_{10}(S) = \log_{10}(c) + z\log_{10}(A) \times isolationTime$	8	-344.29	25.22	0	1	180.26
$\log_{10}(S) = \log_{10}(c) + z\log_{10}(A) + elevation$	7	-344.16	25.34	0	1	179.17
$\log_{10}(S) = \log_{10}(c) + z\log_{10}(A) + speciesPool$	7	-343.80	25.71	0	1	178.99
$\log_{10}(S) = \log_{10}(c) + z\log_{10}(A) \times speciesPool$	8	-343.57	25.94	0	1	179.90
$\log_{10}(S) = \log_{10}(c) + z\log_{10}(A) \times elevation$	8	-342.49	27.02	0	1	179.36
$\log_{10}(S) = \log_{10}(c) + z\log_{10}(A) + latitude$	7	-342.41	27.10	0	1	178.29

SI Dataset S1 (species_records.csv)

Data on incidence records of bird species in forest remnants, including species-level information on forest dependency and conservation status.

SI Dataset S2 (sites_data.csv)

Data on forest remnants, including response and predictor variables used in the analysis. The associated R code is available in the Supporting Information.

References

1. M Anciães, M Marini, The effects of fragmentation on fluctuating asymmetry in passerine birds of brazilian tropical forests. *J. Appl. Ecol.* **37**, 1013–1028 (2000).
2. L dos Anjos, L Zanette, EV Lopes, Effects of fragmentation on the bird guilds of the atlantic forest in north paraná, southern brazil. *Ornitol. Neotrop.* **15**, 137–144 (2004).
3. LWL de Araújo, Master's thesis (Universidade Federal de Alagoas, Maceió, Brazil) (2013).
4. M Aurélio-Silva, M Anciães, LMP Henriques, M Benchimol, CA Peres, Patterns of local extinction in an amazonian archipelagic avifauna following 25 years of insularization. *Biol. Conserv.* **199**, 101–109 (2016).
5. AA Bispo, Ph.D. thesis (Universidade Estadual Paulista, São José do Rio Preto, Brazil) (2010).
6. AL Boesing, E Nichols, JP Metzger, Biodiversity extinction thresholds are modulated by matrix type. *Ecography* **41**, 1520–1533 (2018).
7. AS Bueno, SM Dantas, LMP Henriques, CA Peres, Ecological traits modulate bird species responses to forest fragmentation in an amazonian anthropogenic archipelago. *Divers. Distributions* **24**, 387–402 (2018).
8. AS Bueno, CA Peres, The role of baseline suitability in assessing the impacts of land-use change on biodiversity. *Biol. Conserv.* **243**, 108396 (2020).
9. E Carrara, et al., Impact of landscape composition and configuration on forest specialist and generalist bird species in the fragmented lacandona rainforest, mexico. *Biol. Conserv.* **184**, 117–126 (2015).
10. O Claessens, Diversity and guild structure of the petit saut bird community. *Rev. Ecol.* **57**, 77–102 (2002).
11. GC Daily, PR Ehrlich, GA Sánchez-Azofeifa, Countryside biogeography: Use of human-dominated habitats by the avifauna of southern costa rica. *Ecol. Appl.* **11**, 1–13 (2001).
12. FD Dami, GS Mwansat, SA Manu, The effects of forest fragmentation on species richness on the obudu plateau, south-eastern nigeria. *Afr. J. Ecol.* **51**, 32–36 (2013).
13. PF Develey, Ph.D. thesis (Universidade de São Paulo, São Paulo, Brazil) (2004).
14. DP Edwards, et al., Wildlife-friendly oil palm plantations fail to protect biodiversity effectively. *Conserv. Lett.* **3**, 236–242 (2010).
15. K Feeley, Analysis of avian communities in lake guri, venezuela, using multiple assembly rule models. *Oecologia* **137**, 104–113 (2003).
16. TW Gillespie, H Walter, Distribution of bird species richness at a regional scale in tropical dry forest of central america. *J. Biogeogr.* **28**, 651–662 (2001).
17. JH Hatfield, et al., Mediation of area and edge effects in forest fragments by adjacent land use. *Conserv. Biol.* **34**, 395–404 (2020).
18. LMP Henriques, S Dantas, LB Santos, AS Bueno, CA Peres, Avian extinctions induced by the oldest amazonian hydropower mega dam: evidence from museum collections and sighting data spanning 172 years. *PeerJ* **9**, e11979 (2021).
19. LH Holbech, The implications of selective logging and forest fragmentation for the conservation of avian diversity in evergreen forests of south-west ghana. *Bird Conserv. Int.* **15**, 27–52 (2005).
20. GJ Irving, PD Round, T Savini, AJ Lynam, GA Gale, Collapse of a tropical forest bird assemblage surrounding a hydroelectric reservoir. *Glob. Ecol. Conserv.* **16**, e00472 (2018).
21. FP Jensen, AP Tøttrup, KD Christensen, The avifauna of coastal forests in southeast tanzania. *Scopus* **25**, 1–22 (2005).
22. FP Jensen, L Dinesen, LA Hansen, DC Moyer, EA Mulungu, Bird species richness in the montane evergreen forests of the udzungwa mountains, tanzania. *Scopus* **40**, 39–49 (2020).
23. KM Jyothi, PO Nameer, Birds of sacred groves of northern kerala, india. *J. Threat. Taxa* **7**, 8226–8236 (2015).
24. UG Kormann, et al., Primary rainforest amount at the landscape scale mitigates bird biodiversity loss and biotic homogenization. *J. Appl. Ecol.* **55**, 1288–1298 (2018).
25. MM Krügel, L dos Anjos, Bird communities in forest remnants in the city of maringá, paraná state, southern brazil. *Ornitol. Neotrop.* **11**, 315–330 (2000).
26. JR Lasky, TH Keitt, Abundance of panamanian dry-forest birds along gradients of forest cover at multiple scales. *J. Trop. Ecol.* **26**, 67–78 (2010).
27. AC Lees, CA Peres, Avian life-history determinants of local extinction risk in a hyper-fragmented neotropical forest landscape. *Animal Conserv.* **11**, 128–137 (2008).
28. T Luypaert, et al., A framework for quantifying soundscape diversity using hill numbers. *Methods Ecol. Evol.* **13**, 2262–2274 (2022).

29. S Manu, W Peach, W Cresswell, The effects of edge, fragment size and degree of isolation on avian species richness in highly fragmented forest in west africa. *Ibis* **149**, 287–297 (2007).
30. MA Marini, Effects of forest fragmentation on birds of the cerrado region, brazil. *Bird Conserv. Int.* **11**, 13–25 (2001).
31. AC Martensen, MC Ribeiro, C Banks-Leite, PI Prado, JP Metzger, Associations of forest cover, fragment area, and connectivity with neotropical understory bird species richness and abundance. *Conserv. Biol.* **26**, 1100–1111 (2012).
32. CD Mendenhall, A Shields-Estrada, AJ Krishnaswami, GC Daily, Quantifying and sustaining biodiversity in tropical agricultural landscapes. *Proc. Natl. Acad. Sci.* **113**, 14544–14551 (2016).
33. RP Moore, WD Robinson, IJ Lovette, TR Robinson, Experimental evidence for extreme dispersal limitation in tropical forest birds. *Ecol. Lett.* **11**, 960–968 (2008).
34. JC Morante-Filho, D Faria, E Mariano-Neto, J Rhodes, Birds in anthropogenic landscapes: The responses of ecological groups to forest loss in the brazilian atlantic forest. *PLoS ONE* **10**, e0128923 (2015).
35. A Nasruddin-Roshidi, et al., Recovery of bird communities following the construction of a large-scale hydroelectric dam. *Ecol. Process.* **10**, 30 (2021).
36. HS Oliveira, SF Gouveia, J Ruiz-Esparza, SF Ferrari, Fragment size and the disassembling of local bird communities in the atlantic forest: A taxonomic and functional approach. *Perspectives Ecol. Conserv.* **18**, 304–312 (2020).
37. R Ribon, JE Simon, GT de Mattos, Bird extinctions in atlantic forest fragments of the viçosa region, southeastern brazil. *Conserv. Biol.* **17**, 1827–1839 (2003).
38. V Ruiz-Gutiérrez, EF Zipkin, AA Dhondt, Occupancy dynamics in a tropical bird community: unexpectedly high forest use by birds classified as non-forest species. *J. Appl. Ecol.* **47**, 621–630 (2010).
39. LF Silveira, F Olmos, AJ Long, Birds in atlantic forest fragments in north-east brazil. *Cotinga* **20**, 32–46 (2003).
40. PC Stouffer, EI Johnson, RO Bierregaard, TE Lovejoy, Understory bird communities in amazonian rainforest fragments: Species turnover through 25 years post-isolation in recovering landscapes. *PLoS ONE* **6**, e20543 (2011).
41. A Uezu, JP Metzger, Vanishing bird species in the atlantic forest: relative importance of landscape configuration, forest structure and species characteristics. *Biodivers. Conserv.* **20**, 3627–3643 (2011).
42. W Ulrich, L Lens, JA Tobias, JC Habel, Contrasting patterns of species richness and functional diversity in bird communities of east african cloud forest fragments. *PLoS ONE* **11**, e0163338 (2016).
43. Y Wang, et al., No evidence for the small-island effect in avian communities on islands of an inundated lake. *Oikos* **121**, 1945–1952 (2012).
44. R Wethered, MJ Lawes, Nestedness of bird assemblages in fragmented afromontane forest: the effect of plantation forestry in the matrix. *Biol. Conserv.* **123**, 125–137 (2005).
45. DL Yong, et al., Do insectivorous bird communities decline on land-bridge forest islands in peninsular malaysia? *J. Trop. Ecol.* **27**, 1–14 (2011).
